# Extracellular Vesicles of Adipose-Derived Stem Cells Promote the Healing of Traumatized Achilles Tendons

**DOI:** 10.3390/ijms222212373

**Published:** 2021-11-16

**Authors:** Shih-Heng Chen, Zhi-Yu Chen, Ya-Hsuan Lin, Shih-Hsien Chen, Pang-Yun Chou, Huang-Kai Kao, Feng-Huei Lin

**Affiliations:** 1 Institute of Biomedical Engineering, College of Medicine and College of Engineering, National Taiwan University, Taipei 10617, Taiwan; shihheng@mac.com (S.-H.C.); d79010340217@gmail.com (Z.-Y.C.); d9823007@gmail.com (S.-H.C.); 2 Department of Plastic and Reconstructive Surgery, Chang Gung Memorial Hospital, Chang Gung University and Medical College, Taoyuan 33305, Taiwan; Yhlinlf@gmail.com (Y.-H.L.); chou.asapulu@gmail.com (P.-Y.C.); 3 Division of Biomedical Engineering and Nanomedicine Research, National Health Research Institutes, Miaoli 35053, Taiwan

**Keywords:** adipose-derived stem cells, extracellular vesicles, tendon healing, tendon regeneration

## Abstract

Healing of ruptured tendons remains a clinical challenge because of its slow progress and relatively weak mechanical force at an early stage. Extracellular vesicles (EVs) derived from mesenchymal stem cells (MSCs) have therapeutic potential for tissue regeneration. In this study, we isolated EVs from adipose-derived stem cells (ADSCs) and evaluated their ability to promote tendon regeneration. Our results indicated that ADSC-EVs significantly enhanced the proliferation and migration of tenocytes in vitro. To further study the roles of ADSC-EVs in tendon regeneration, ADSC-EVs were used in Achilles tendon repair in rabbits. The mechanical strength, histology, and protein expression in the injured tendon tissues significantly improved 4 weeks after ADSC-EV treatment. Decorin and biglycan were significantly upregulated in comparison to the untreated controls. In summary, ADSC-EVs stimulated the proliferation and migration of tenocytes and improved the mechanical strength of repaired tendons, suggesting that ADSC-EV treatment is a potential highly potent therapeutic strategy for tendon injuries.

## 1. Introduction

Tendons are connective tissues that connect muscles to bone and transmit force from muscle to skeleton, generate range of motion in joints, and maintain tension in positioning [1]. Tendons are composed of highly ordered type I collagen fibers as their major extracellular matrix (60–85% dry weight) and a relatively small amount of cellular components, mainly tenocytes [2]. Tendon injury is a common trauma that may cause morbidities such as adhesion and reduced range of motion in joints because of its prolonged healing time. This also results in limitations in working ability and compromised quality of life. The prolonged healing of tendons stems from its characteristics, including hypovascularity and low metabolic activity, which are designed for tolerance of hypoxia during continuous exercise [3]. After trauma, the prolonged inflammatory phase of the healing process can induce fibroblast activity and result in excessive scar formation and subsequent tendon adhesion, which would compromise the functional outcome of tendon repair [4]. Currently, promoting tendon healing and reducing downtime remains a major clinical challenge [5].

Mesenchymal stem cells (MSCs) possess multi-lineage differentiation ability, and thus promote tissue regeneration and functional recovery [6]. These characteristics make MSCs widely investigated in regenerative medicine, thus enlightening therapeutic strategies for restoring the function of tissues and organs. To date, multiple studies have shown the efficacy of MSC-based therapy for the promotion of tendon healing by either direct injection of MSCs directly into injured sites or the implementation of MSC-loaded scaffolds during surgical repair [7,8,9,10]. However, for a long time, direct cell therapy has still faced critical challenges, including safety concerns, potential tumorgenicity, preservation, and cell expansion cost. Although MSCs have the ability of tenogenic differentiation and increase the effectiveness of tendon healing [11], many studies have indicated that the therapeutic effects of non-tendon tissue MSCs are predominantly due to the contribution of empowering resident cells via intercellular communication or paracrine effects [12].

MSCs can be isolated from various tissues, while bone marrow and adipose tissue are more commonly used in clinical trials [13]. However, in clinical scenarios, harvesting bone marrow-derived MSCs is relatively more invasive than harvesting adipose-derived stem cells (ADSCs). Moreover, evidence has shown that ADSCs represent an alternative stem cell source in tendon healing compared to bone marrow-derived mesenchymal stem cells (BMSCs) [14,15]. Thus, using ADSCs as the source in regeneration therapy would offer the advantages of easier harvest and abundant tissue source compared to BMSCs [16]. Studies have shown that MSCs modulate inflammation through the release of cytokines and induce tissue growth through the release of growth factors [17,18,19]. It is believed that MSCs promote tissue regeneration via paracrine factors and bring the MSCs into a wider field of therapeutic measurements [19,20], rather than local proliferation of implanted stem cells [21,22,23]. In addition, cell-based therapy still encounters challenges, including immune compatibility, medical ethical issues, and possible transmission of diseases during the process of cell expansion and transportation. Thus, the application of paracrine effects from these MSCs has become another modality to treat various diseases or to promote tissue regeneration, such as ischemic heart disease, acute lung injury, or traumatic brain injury [23,24,25].

Extracellular vesicles (EVs) are membranous vesicles with small size (30–1000 nm) that contain mediators for cell-to-cell communication that transport information to recipient cells and influence various cell functions [26]. EVs affect cells through a paracrine effect [27,28,29]. EVs derived from MSCs have been demonstrated to promote tissue regeneration and functional restoration in various organs, including the heart, kidney, lung, liver, epithelium, muscle, and connective tissue [30]. For example, MSC-derived exosomes, a subgroup of EVs, have been shown to increase re-epithelialization by inducing cell proliferation [18] and enhance the survival of fat grafts by promoting angiogenesis and modulating early inflammation [31]. In contrast to cell therapy, EVs provide a cell-free modality that bypasses the doubt of cell therapy, including tumorigenicity, immune compatibility, and infection [32]. The perspectives of application, easy storage, mass production from certified cell lines, and reduction of stem cell expansion time and cost make EVs promising pharmaceutical agents.

The goal of this study was to elucidate the tendon regenerative ability of ADSC-derived EVs in vitro and in a rabbit Achilles tendon transection/repair model. In this study, the effects of ADSC-EVs on the proliferation and migration of tenocytes in vitro and on the biomechanical strength and protein expression of repaired tendons in vivo were investigated.

## 2. Results

### 2.1. Characterization of ADSC-EVs

**Figure 1:**
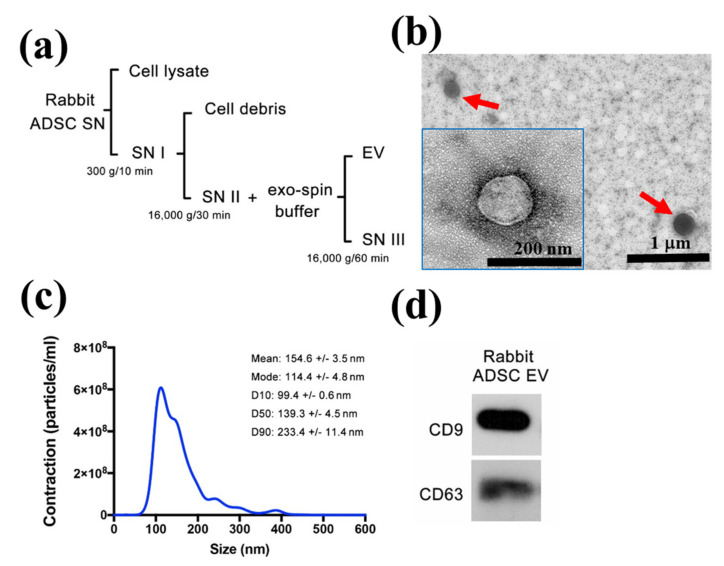
Preparation and characterization of ADSC-EVs. (**a**) Flowchart demonstrating the isolation of EVs from the culture supernatant (SN) of ADSCs by differential centrifugation. (**b**) Rounded morphology and bilayer structure of ADSC-EVs was observed using TEM. (**c**) Size distribution was analyzed using NTA. (**d**) Western blotting to detect CD9 and CD63, specific surface markers of EVs.

The protocol for isolating ADSC-EVs is shown in Figure 1a. To clarify the size distribution and shape, isolated ADSC-EVs were analyzed using transmission electron microscopy (TEM) and nanoparticle tracking analysis (NTA). TEM (Figure 1b) showed that EVs appeared as round vesicular shapes with a bilayer structure. NTA revealed the size distribution of EVs, which ranged from 99.4 nm to 233.4 nm with mean diameter of 154.6 nm (Figure 1c). The size and shape are consistent with those of previous studies [26,33,34]. In the aspect of EVs specific markers, the expression of Cluster of differentiation 9 (CD9) and CD63 were detected by Western blot (Figure 1d). All of these characteristics required for the definition of EVs were consistent with those of previous studies [34,35]. In addition, we measured the protein content of EVs using the Bradford assay. The mean protein concentration of the harvested ADSC-EVs was 4.20 ± 1.85 × 10^3^ μg/mL.

### 2.2. ADSC-EVs Promote the Proliferation of Tenocyte

**Figure 2:**
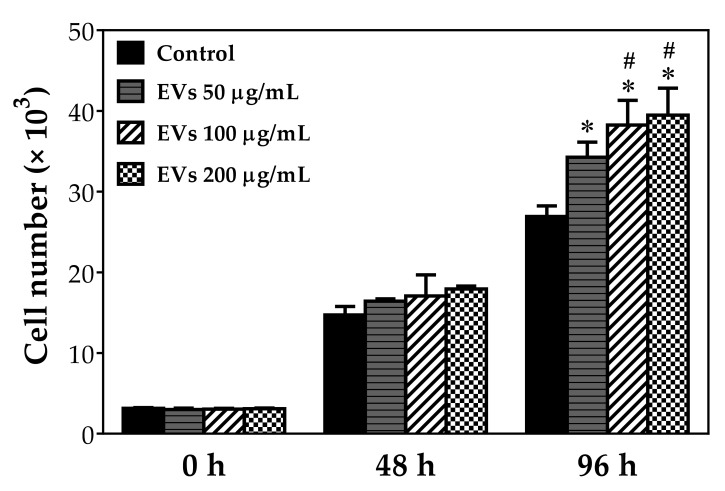
ADSC-EVs promote tenocyte proliferation. Tenocytes isolated from Achilles tendons of rabbits were treated with different concentrations of ADSC-EVs (50, 100, and 200 μg/mL) for 96 h and evaluated using the WST-1 assay at 0, 48, and 96 h. All experiments were repeated six times. * *p* < 0.05 compared with control group # *p* < 0.05 compared with 50 μg/mL EVs-treated group.

To investigate the interaction of ADSC-EVs with tenocytes, we exposed tenocytes to three different protein concentrations of EVs (50 μg/mL, 100 μg/mL, and 200 μg/mL) for 96 h and then analyzed the proliferative activity of treated tenocytes (Figure 2). Compared to the control group, the results demonstrated that all three groups of EVs had significantly increased proliferation up to 28.7% (*p* < 0.05), 44.2% (*p* < 0.05) and 49.1% (*p* < 0.05) at 96 h, respectively. Furthermore, comparison between each group treated with different concentrations showed that tenocytes treated with 100 μg/mL or 200 μg/mL concentration had significantly more proliferation than the 50 μg/mL group at 96 h (12.2% and 15.9% increase, respectively; both *p* < 0.05). Although there was no significant difference between the 100 μg/mL and 200 μg/mL groups, the 200 μg/mL group still had slightly increased cell proliferation compared to the 100 μg/mL group. These results not only implied that ADSC-EVs effectively promoted the proliferation of tenocytes, but also indicated that its effect was dose-dependent, with a plateau likely around 200 μg/mL protein concentration.

### 2.3. ADSC-EVs Improve Tenocyte Migration Ability

**Figure 3:**
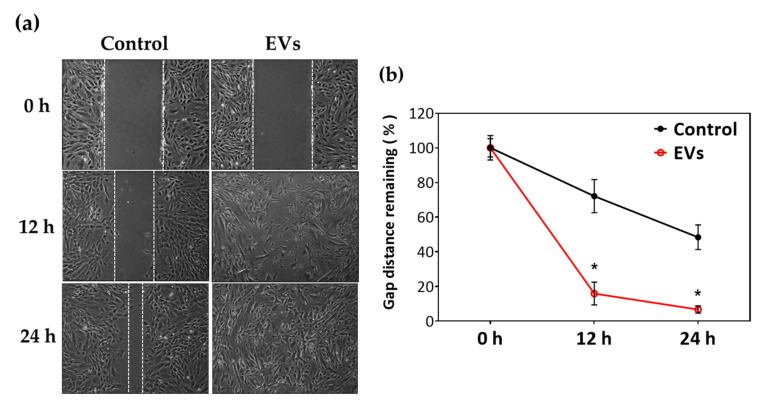
ADSC-EVs promote tenocyte migration. Effects of 100 μg/mL ADSC-EVs on migration of tenocytes compared with that of control group were determined by gap-closing assay at 0, 12, and 24 h. All experiments were repeated five times. (**a**) Representative photomicrographs. (**b**) Quantification of remaining gap area using image J. * *p* < 0.05 compared with control group.

To elucidate the influence of EVs on the migration of tenocytes, a gap-closure migration assay was performed. The results (Figure 3) were analyzed by Image J and presented as the percentage of remaining gap area at 12 and 24 h by comparing the gap area at 0 h as 100%. Compared to the control group, EV-treated tenocytes had a significantly reduced gap area at both 12 h (71.2 ± 9.6% compared to 15.9 ± 6.6%; *p* < 0.05) and 24 h (48.3 ± 7.1% compared to 6.7 ± 2.1%; *p* < 0.05). These results indicated that ADSC-EVs significantly promoted the migration of tenocytes.

### 2.4. ADSC-EVs Improve the Mechanical Strength of Repaired Tendon In Vivo

**Figure 4:**
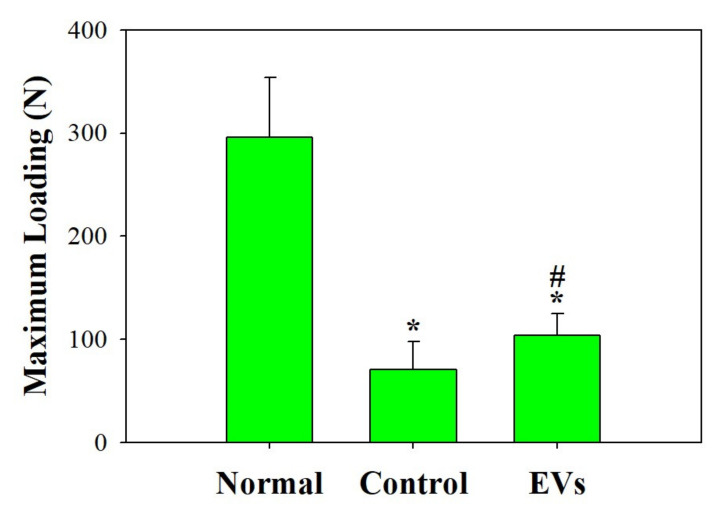
Quantitative evaluation of biomechanical properties of repaired tendons. The maximum loading force was defined as the minimum stretching force required to tear apart the tendon. The breaking force for EV-treated tendons was 1.47-fold higher than that of the control group. * *p* < 0.05 compared with normal group; # *p* < 0.05 compared with control group.

**Figure 5:**
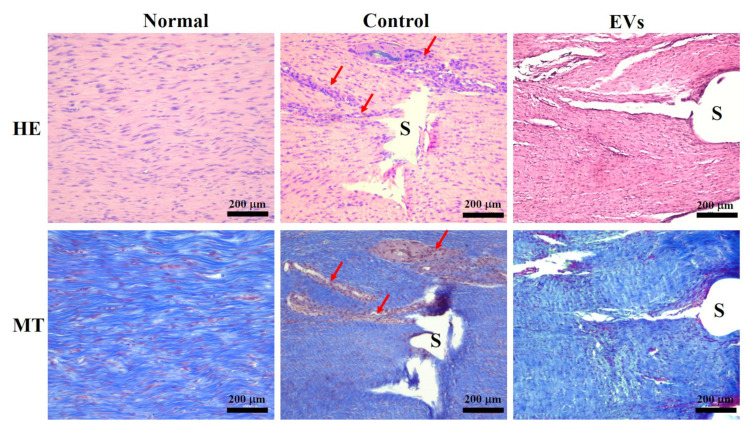
Histological analysis of healing tendons at week 4 using H&E (HE) and Masson’s trichrome (MT) staining. The interface of repaired tendons is visible in photomicrographs. Red arrows indicate the hypercellularity of control group compared with that of EV-treated group and healthy tendons. S: suture.

Next, to investigate whether ADSC-EVs promote regeneration of tendons and have benefits on mechanical properties in vivo, we injected ADSC-EVs in the repaired Achilles tendons in a rabbit model and compared them with the control group, which received tendon repair and PBS injection in the repaired tendon. To observe the mechanical properties of the repaired tendon, the breaking force was measured and compared between the EV-treated and control groups. Figure 4 shows that the breaking force was significantly higher in the EV-treated group (104 ± 21 N; *p* < 0.05) than in the control group (71 ± 27 N), implying that EV-treated tendons healed better and required more strength to break. No gross adverse effects were observed during the harvest of the repaired tendons in any of the groups. For histological analysis, sections of the tendon were stained with H&E and Masson’s trichrome stain (Figure 5). Under microscopic observation, the EV-treated group had more organized and aligned collagen fibers with less inflammatory cell infiltration compared to the control group. In contrast, the control group had more neutrophil infiltration than the EV-treated group. These results indicate that ADSC-EV injection promotes tendon regeneration and may reduce inflammation, which is beneficial for tendon repair in clinical scenarios.

### 2.5. ADSC-EVs Increase the Expression of Tenomodulin, Biglycan and Decorin In Vivo

**Figure 6:**
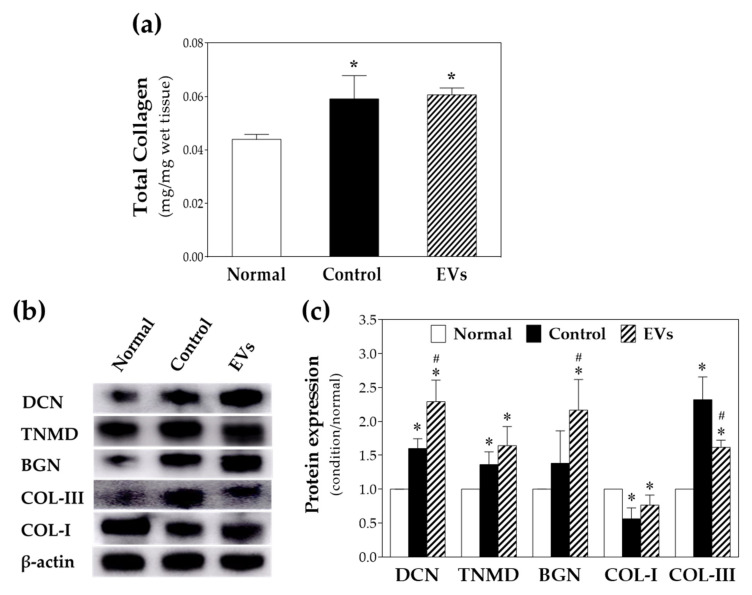
(**a**) Total collagen assay and (**b**) Western blotting for detection of decorin (DCN), tenomodulin (TNMD), biglycan (BGN), collagen I (CoL-I), collagen III (CoL-III) and beta-actin. (**c**) Quantification of Western blot. * *p* < 0.05 compared with normal group; # *p* < 0.05 compared with control group.

To evaluate the regeneration potential of ADSC-EVs on repaired tendons in vivo, total collagen amount was measured by collagen assay, while collagen I, collagen III, decorin, tenomodulin, and biglycan were quantitatively evaluated by Western blotting with β-actin as an internal control (Figure 6). Total collagen significantly increased in both the EV-treated and control groups but not in normal tendons, which reflects the necessity of collagen formation after tendon injury and during its healing process. Collagen III stands for a less mature extracellular composition [36]; in contrast, collagen I stands for a more mature status [37]. The presence of tenomodulin represents the maturation of the repaired tendon [38], and higher tenomodulin level also implies higher proliferation and migration ability of tenocytes [39]. Elevated biglycan levels indicate tendon formation by modulating collagen organization and maturation [40] in addition to increased migration ability of cells [41]. As shown in Figure 6c, the quantitative analysis showed that the ADSC-EVs treated group had relatively higher expression of collagen I and significantly lower expression of collagen III compared with the surgical control group. Furthermore, expression of decorin and biglycan was significantly upregulated in the ADSC-EV-treated group compared with the control group. These differences demonstrate that ADSC-EVs potentially increase the expression of decorin, collagen I, tenomodulin, and biglycan, indicating that the ADSC-EVs possessed a regenerative effect in injured tendons with a more mature collagen expression and tenocyte activity, which also echoes the biomechanical findings that EV-treated tendons had higher breaking force than the control group (Figure 4). The findings that EV-treated group presented significantly higher biglycan level and relatively higher tenomodulin level also reflects the findings of the tenocyte migration test in vitro (Figure 3).

## 3. Discussion

The healing process of the injured tendon undergoes three phases: inflammation, proliferation, and remodeling, and these phases overlap with each other. After injury, inflammatory responses occur, and cytokines and growth factors induce angiogenesis, stimulate tenocyte proliferation, and generate collagen III. Following progress, tenocytes and collagen organize and align, and higher synthesis of collagen I than collagen III leads to changing proportions of collagen fibers in the healing tendon. Several factors limit tendon healing. Overt and inappropriate inflammatory responses in the early phase and immobilization after repair result in granulation tissue formation and adhesion [3]. In addition, disorganized tissue and different extracellular matrix components in injured tendons cause unreachable mechanical loading properties compared to native tendons [5,42]. Thus, controlling inflammation and regulating cell growth, alignment, and components is important in the tendon regeneration field. Previous studies have shown the effectiveness and practical use of water-borne polyurethane nanofibrous membranous materials and other materials as barriers to reduce the degree of peritendinous adhesion in a rabbit model under safety [43,44]. On the other aspect of restoring the components and mechanical properties of injured tendons, there are still limited studies that report approaches that promote tissue regeneration, making tendon tissue regeneration a challenge in current therapeutic settings.

EVs have been generally accepted as a modality of cell-to-cell communication for MSCs to pass the signal to recipient cells via various pathways [33]. Previous studies have indicated that EVs can enhance the healing process of tendon injury [7,45,46,47]. MSC-derived EVs are believed to regulate early inflammation in tendon repair and thus have therapeutic potential in reducing fibrotic scar formation, thereby facilitating tendon healing [4,45,46,48,49]. Moreover, bone marrow MSC-derived EVs promote tendon healing by facilitating the proliferation and migration of tendon cells [50], and similar results were shown in tendon stem cell-derived exosomes [51]. However, limited research has proved the potency of ADSC-EVs in promoting tendon cell proliferation and migration. In addition, the restoration of biomechanical strength and the tissue composition of EV-treated tendons in vivo still lacks evidence and requires further investigation. In our study, we purified EVs from ADSCs of New Zealand white rabbits via differential centrifugation and determined their characteristics and protein concentration, as described in a previous study [33]. Because there is little evidence regarding the optimal concentration of EVs to promote tendon regeneration, different concentrations of EVs were tested in the current study to elucidate the most effective concentration of ADSC-EVs for promotion of tenocyte activity and for further in vivo studies.

The proliferation and migration of tenocytes are believed to play an important role in tendon healing [52] and are related to the strength of the healed tendons [53,54,55,56]. In our previous study, human tenocytes treated with polysaccharides extracted from *Bletilla striata* demonstrated better migration and enhanced proliferation via activation of MEK/ERK1/2 and PI3K/Akt signaling pathways, as well as in the secretion of extracellular matrix [57]. Similarly, bone marrow MSC-derived conditioned medium enhances the proliferation and migration of tenocytes, leading to improved healing of tendons [58]. Moreover, evidence showed that ADSC-derived microvesicles enhance wound healing via similar pathways [59]. While the effect of ADSC-EVs on tenocytes remains uncertain in the literature, we successfully proved that ADSC-EVs could promote not only tenocyte proliferation but also migration. The effect of ADSC-EVs in the present study revealed that cell proliferation increased significantly at 96 h, which was as equally effective as a previous study using exosomes derived from bone marrow MSCs [50]. In addition, we demonstrated a dose-dependent effect of ADSC-EVs on the proliferation of tenocytes, and an optimal concentration of 100 μg/mL was identified. The significant improvement in the migration of tenocytes after ADSC-EV treatment at early 12 h also implies that ADSC-EVs activated the signal pathway promptly to promote the migration of tenocytes.

Mechanical strength is an indicator of tendon healing and functional outcomes [43,44,60,61]. A recent study showed improved biomechanical properties in a repaired rotator cuff model with treatment with exosome-embedded fibrin sealant [62]. However, the effects of ADSC-EVs on repaired tendons have rarely been discussed. In this study, we proved that EV-treated tendons had a 1.47-fold higher breaking force than the surgical control group. Histological staining showed better alignment of collagen fibers and less neutrophil infiltration in the EV-treated group, which indicated that EVs might improve the structural arrangement of the repaired tendon and reduce the inflammation after trauma.

In the present study, the collagen composition and protein expression of ADSC-EV-treated tendons, including collagen I, collagen III, biglycan, decorin, and tenomodulin, were quantified by Western blotting. Collagen I is the predominant component of the extracellular matrix (ECM) in normal tendons and possesses major mechanical strength [63]. Studies have shown that collagen III is upregulated in both early stages of tendon healing and is related to tendon scarring and adhesion [64]. Our results revealed that the ADSC-EVs treated group expressed relatively more collagen I and significantly less collagen III compared to the surgical control group. In addition, compared with the normal healthy tendon group, the ADSC-EVs group had only a 24% difference of the collagen I level, indicating that ADSC-EVs modulated the composition of collagen closer to normal healthy tendons and increased mechanical strength after tendon repair. Tenomodulin is a widely accepted tendon-specific marker in tendon maturation and is related to the prevention of tendon scarring since the early stage [38]. Loss of expression of tenomodulin impairs the accumulation of tendon collagen I and contractility of the ECM [65,66]. On the contrary, higher expression of tenomodulin would benefit tendon healing by promoting tenocyte differentiation and proliferation [11,67,68]. The results of this study showed that the ADSC-EVs treated group expressed relatively higher tenomodulin content, implying that ADSC-EVs have the ability to promote tenocyte proliferation and ECM remodeling, which also reflects the results of tenocyte proliferation after treatment with EVs in our in vitro study. In addition, biglycan, a small leucine-rich repeat proteoglycan, plays an important role in regulating collagen organization, maturation, and tenocyte differentiation and migration [41]. The absence of biglycan in the early healing process adversely affects the outcome of tendon repair [69]. Another study found that additional supplementation of biglycan and decorin to the peritenon and tendon proper in vitro improved the mechanical properties of tendons [40]. In our study, ADSC-EVs were found to upregulate the expression of biglycan compared with the surgical control group, which implied the potential for promotion of tendon healing and regeneration. The upregulation of biglycan and tenomodulin level in the EV-treated group also reflects the findings of tenocyte migration in vitro.

The application of ADSC-EVs in regenerative medicine has emerged in recent years after discovering that the beneficial effect of stem cell therapy was significantly related to paracrine action [70,71,72]. The easy availability of adipose tissue and high proliferation potential makes ADSCs more feasible than other sources of MSCs, such as bone marrow [73]. EVs provide a cell-free therapeutic option and overcome some challenges of MSC therapy in regenerative medicine, such as immune compatibility, tumorigenicity, and emboli formation [74]. Previous studies have proved the benefits of BMSC-EVs in tendon healing and restoration of mechanical strength [50,75,76]. However, not all EVs are created equal in their regenerative potential [77,78], and the effects of ADSC-EVs on tendon healing both in vitro and in vivo have not yet been elucidated. This study demonstrated that ADSC-EVs promoted the proliferation and migration of tenocytes, enhanced the mechanical properties of repaired tendons, and improved collagen alignment, composition, and maturation in repaired tendons.

There are still limitations in this study that require further investigation. First, we found that ADSC-EVs promoted the proliferation of tenocytes in a dose-dependent manner in vitro, and whether this dose-dependent pattern is present in the in vivo model remains to be studied in future. However, the EV concentration applied in our study still demonstrated the effectiveness of ADSC-EVs in tendon repair. Second, more time points (such as 2 or 6 weeks after tendon repair) could be evaluated in addition to the 4 weeks in vivo, including the mechanical strength and protein expression of repaired tendons, to understand if there are sequential changes in tendon strength and tendon-specific markers at different stages of tendon healing. Finally, the contents of ADSC-EVs should be studied to determine the effective cargo in the EVs.

## 4. Materials and Methods

### 4.1. Animal Experiments/Ethics

All animal experimental designs were approved by the Animal Research Committee of Chang-Gung Memorial Hospital and complied with the Guide for the Care and Use of Laboratory Animals with project identification code: IUCAC-2020072203 and date of approval from 1 January to 31 December 2021.

### 4.2. Harvest of ADSCs

The ADSCs were isolated from the fat pads of female New Zealand white rabbits (National Laboratory Animal Breeding and Research Center, Taipei, Taiwan). After harvest of fat pads, the adipose tissues were irrigated in sterile phosphate-buffered solution (PBS; HyClone, Logan, UT, USA, cat. SH30256.01) and fragmented with tenotomies. The fragmented adipose tissue was digested using 1 mg/mL collagenase (Gibco, Grand Island, NY, USA, cat. 17100-017) for 40 min at 37 °C with shaking, followed by neutralization with an equal volume of 5% bovine serum album (BSA; Sigma-Aldrich, St. Louis, USA, cat. A2153) in PBS. The undigested tissues were removed by using a 147 µm nylon mesh and the filtrate was centrifuged at 200 *g* for 6 min at 4 °C, followed by collection of the pellet and resuspension in DMEM medium (Gibco, Life Technologies, Carlsbad, CA, USA, cat. 31600-034) with 1% BSA, with addition of erythrocyte lysis buffer (Beckman Coulter, Miami, FL, USA, Cat. IM3630d) on ice for 10 min and centrifugation at 500 *g* for 8 min at 4 °C. The isolated cells were seeded and expanded in medium with DMEM/F12 (Gibco, Life Technologies, Carlsbad, CA, USA, cat. 12500-062) containing 10% fetal bovine serum (FBS; STEMCELL Technologies, Vancouver, Canada, cat. 05420) and supplemented with 1% (*v*/*v*) penicillin/streptomycin (10,000 U/mL penicillin and 10,000 µg/mL streptomycin, Gibco BRL, Grand Island, NY, USA, cat. 15140-122) and 2 mM L-glutamine (Gibco BRL, Grand Island, NY, USA, cat. 25030-081). All cells were cultured at 37 °C in a humidified atmosphere with 5% CO_2_.

### 4.3. Preparation of ADSC Derived EVs

For the preparation of ADSC-derived EVs, the culture medium was replaced with exosome-free Dulbecco’s Modified Eagle Medium (DMEM; Gibco Invitrogen/Life Technologies, Carlsbad, CA, USA) supplemented with 10% fetal bovine serum (FBS; Gibco Invitrogen/Life Technologies, Carlsbad, CA, USA), 2 mM l-glutamine and 100 U penicillin/100 U streptomycin when the cells met 70–80% confluent. The supernatants from cells grown in conditioned medium including 5% exosome-depleted FBS (Thermo Fisher Scientific, Waltham, MA, USA) were collected after 72 h. Extracellular vesicles were isolated via differential centrifugation at 4 °C in the following order: first centrifugation at 3000 *g* for 10 min to remove suspended cells, then 16,000 g for 30 min to remove cell debris, and finally centrifugation at 16,000 *g* for 60 min with exo-spin buffer (Cell Guidance Systems, St. Louis, MO., USA). The EV pellets were re-suspended in PBS and stored at −80 °C for subsequent experiments (Figure 1a).

### 4.4. Characterization of ADSC-Derived EVs

The extracellular vesicles were analyzed by transmission electron microscopy (JEOL 1200EX, Tokyo, Japan) and nanoparticle tracking analysis (NanoSight LM10, Amesbury, United Kingdom) to reveal their ultrastructure and size distribution, respectively. The protein markers including CD9 and CD63 (Biolegend, San Diego, CA, USA) were determined by Western blot. The protein concentration in EVs was analyzed using the Bradford assay.

### 4.5. Tenocyte Proliferation Assay

Primary tenocytes were isolated from Achilles tendons of New Zealand white rabbits (2 months old). After harvesting the Achilles tendons, the tendons were divided into pieces measuring 1~2 mm in size, and digested in 5 mL of DMEM containing 1 mg/mL of collagenase and 0.25% trypsin solution. After 30 min at 37 °C, tendons were incubated for 30 min at room temperature with stirring. The medium was replaced with a solution supplemented with 10% FBS and 0.25 mg/mL collagenase and incubated in 37 °C overnight to collect the suspension. A 0.3 μm filter was used to filter the suspension and the filtrate was centrifuged at 1500 g at 4 °C for 5 min. The cell pellet was suspended in 10 mL of DMEM supplemented with 10% FBS and 4 μg/mL  gentamicin. The cells were seeded at 2 × 10^6^ cells per 75-cm^2^ flask and grown at 37 °C. The medium was changed the day after seeding and every 2 days. The cells were collected with trypsin when cells met 80% confluency at passage 2–6 and were calculated by cell counter.

For proliferation assay, 3 × 10^4^ collected rabbit tenocytes were seeded in 96-well culture plate and incubated for 8 h. The culture medium was removed and the cells were irrigated twice with fresh PBS. Each well was incubated with different concentrations of EVs (0, 50, 100 and 200 μg/mL) diluted with culture media for 0, 2, and 4 days. The culture medium was removed and the cells were washed twice with PBS. The plates were then incubated with diluted WST-1 (1:9 dilution with conditioned media) for another 1.5 h. The optical density at 450 nm was measured using an ELISA reader, and cell numbers were determined by a calibration curve. All the experimental results were independently repeated six times.

### 4.6. Tenocyte Migration Assay

The efficiency of tenocyte migration was determined through gap-closing assay by the use of two-well silicone culture inserts (Ibidi GmbH, Martinsried, Germany) inset on the transparent dishes (Ibidi GmbH, Martinsried, Germany) without coatings. Tenocytes were seeded in the culture inserts (8 × 10^3^ cells/cm^2^) with 70 μL DMEM containing 20% FBS. After resting for 8 h, a single layer of confluent cells could be noted on the transparent dish. At this point, once the culture inserts were removed, a 500 μm gap was produced in the middle of the dish. Tenocytes were washed with PBS, and then incubated in a serum-free medium at 37 °C for 1 h. The medium was then replaced with EVs (100 μg/mL) diluted with culture media, and tenocytes were left to migrate for 24 h from the medial edges of the 500 μm gap. The migration assays were performed using the control group and EVs group. The migrated tenocytes were observed and captured under bright field microscopy with a camera (Leica QWin, Wetzlar, Germany) from 0 to 24 h. The percentage of remaining area was evaluated using Image J. All results were obtained in five independent experiments.

### 4.7. Animal Study

Rabbits were randomly assigned to the surgical control or EVs group. In brief, the skin of the posterior ankle and distal lower leg was longitudinally incised, and the Achilles tendon was released from the synovial sheath. The Achilles tendon was then transected using a sharp blade, followed by repair with the Modified Kessler four-core technique using 5–0 prolene. Regarding the Modified Kessler repair, the suture was anchored to the tendon at four corners with a single knot, and each tendon had two sets of Modified Kessler sutures, meaning four core sutures passed through the transection surface. In the EV group, EV solution was injected into the repaired Achilles tendon, whereas in the surgical control group, PBS was injected instead. Then the wound was closed primarily and the ankle was immobilized with a cast. Four weeks postoperatively, the rabbits were euthanized, and their Achilles tendons were harvested. The histological sections were stained with H&E and Masson’s trichrome stain. To determine the tendon healing or regenerative effects among different treatment groups, the breaking force of the repaired tendon was measured using a material testing machine. The minimum force required to tear the repaired tendon was defined as the breaking force. Healthy Achilles tendons were also harvested for testing. All results were obtained in six independent experiments.

### 4.8. Protein Expression Analysis of EV-Treated Tendon

#### 4.8.1. Total Collagen Assay

Rabbit Achilles tendons (10 mg) were homogenized with dH2O (100 μL) by μT-12 tissue homogenizer (TAITEC, μT-12, Koshigaya-shi, Japan). A total of 100 μL of 10 N NaOH was added to tissue homogenate and heated at 120 °C for 1 h. Following alkaline hydrolysis, we neutralized residual NaOH by 100 μL 10 N HCl. Total collagen content (hydroxyproline) in rabbit Achilles tendon was determined using a perchlorate-free hydroxyproline assay kit (Biovison, K226-100, Milpitas, CA, USA).

#### 4.8.2. Western Blot

Then, 100 mg of Achilles tendons was mixed with 400 μL tissue protein extraction reagent (ThermoFisher SCIENTIFIC, T-PER^TM^, 78510, Waltham, MA, USA) containing protease inhibitor (Roche, 05892791001, NJ, USA) and phosphatase inhibitor (Roche, 04906837001, NJ, USA). The protein of the treated tendon was extracted following processing of tendons using a tissue homogenizer (TAITEC, μT-12, Nishikata, Japan). To the detection of various proteins related to tendon regeneration, 50 μg of extracted total protein was separated in 10% (300 V, 35 min) SDS–PAGE gels (BIO-RAD, FastCast Acrylamide Kit, 1610183, Irvine, WA, USA) for collagen I and collagen III, or 12% (300 V, 35 min) SDS-PAGE gels for decorin, tenomodulin, biglycan and β-actin for gel electrophoresis, followed by transferring the separated proteins to a PVDF membrane (BIO-RAD, Immum-Blot^®^ PVDF membrances, 1620177, Irvine, WA, USA) at 400 mA for 1 h. Membranes were then blocked using blocking buffer (VISUAL PROTEIN, BlockPRO^TM^ 1 Min Protein-Free Blocking Buffer, BM01-500, Taipei, Taiwan) for 60 s, followed by incubation with anti-collagen I (1:1000, ThermoFisher SCIENTIFIC, MA1-26771, mice monoclonal, Waltham, CA, USA) and III (1:1000, ThermoFisher SCIENTIFIC, MA1-22147, mice monoclonal, Waltham, CA, USA), anti-decorin (1:1000, abcam, ab181456, Cambridge, UK), anti-tenomodulin (1:1000, antibodies, ABIN2783853, Aachen, Germany), anti-biglycan (1:1000, abcam, ab226991, Cambridge, UK), or anti-β-actin (1:1000, abcam, ab8227, Cambridge, UK) overnight at 4 °C. As for the secondary antibodies, membranes were incubated with anti-rabbit (1:10,000, invitrogen, G-21234, Waltham, CA, USA) for β-actin, decorin and tenomodulin or anti-mice (1:10,000, invitrogen, G-21040, Waltham, CA, USA) for biglycan, collagen I and III and conjugated with horseradish peroxidase. Membranes were analyzed using the ECL plus detection system (Amersham Biosciences) using Amersham^TM^ Imager 600 platform (GE Health care, Chalfont Saint Giles, Buckinghamshire, UK). Values were normalized using β-actin as a reference. All experimental results were repeated four times independently.

### 4.9. Statistical Analysis

All experiments were repeated independently at least four to six times with three to five parallel measurements. All data are expressed as mean value ± standard deviation. The statistical differences were tested using either one-way analysis of variance (ANOVA) or Student’s *t*-test, and statistical significance was determined as probability values less than 0.05 (*p* < 0.05).

## 5. Conclusions

In summary, this study indicated that ADSC-EVs promote tenocyte proliferation and migration, and thus possess promising therapeutic potential in tendon healing. In addition, ADSC-EVs improved the mechanical properties of the repaired tendon, modulated the collagen composition and maturation, and promoted tendon regeneration by upregulating tendon-specific markers in surgically repaired tendons. These findings support the clinical value of ADSC-EVs in tendon repair.

## Data Availability

All datasets generated and analyzed during the current study are available from the corresponding authors on reasonable request.
